# Use of the Survivors’ Unmet Needs Survey (SUNS) Framework to Understand the Needs of Colorectal Cancer Survivors in Thailand: A Qualitative Descriptive Study

**DOI:** 10.3390/healthcare13172187

**Published:** 2025-09-01

**Authors:** Cherdsak Duangchan, Sarah Abboud, Rohan D. Jeremiah, Geraldine Gorman, Cherdsak Iramaneerat, Alicia K. Matthews

**Affiliations:** 1 Princess Agrarajakumari Faculty of Nursing, Chulabhorn Royal Academy, Bangkok 10210, Thailand; 2 College of Nursing, University of Illinois Chicago, Chicago, IL 60612, USA; abbouds@uic.edu (S.A.); rjerem@uic.edu (R.D.J.); ggorman@uic.edu (G.G.); 3 Faculty of Medicine Siriraj Hospital, Mahidol University, Bangkok 10700, Thailand; cherdsak.ira@mahidol.ac.th; 4 School of Nursing, Columbia University, New York, NY 10032, USA; akm2238@cumc.columbia.edu

**Keywords:** colorectal neoplasms, cancer survivors, survivorship, developing countries, health services, unmet needs, qualitative research, content analysis

## Abstract

**Background:** A significant body of research has identified ongoing unmet needs among cancer survivors. However, there is limited information about the survivorship experiences of patients in low- and middle-income countries. This study examined the experiences of colorectal cancer (CRC) survivors in Thailand to understand their post-treatment needs and priorities. **Methods**: A qualitative descriptive study using semi-structured interviews was conducted with 24 colorectal cancer survivors purposively recruited from a large university hospital in Bangkok, Thailand. The interviews lasted 60–90 min, were audio-recorded, and transcribed verbatim. The data were analyzed using hybrid content analysis, guided by the five domains of the Survivors Unmet Needs Survey (SUNS): information, emotional health, financial concerns, access and continuity of care, and relationships. **Results:** Participants had a mean age of 57 (SD = 10.9) years. Most were male (58.3%) and diagnosed at a late stage (62.5%). All participants had undergone surgical treatment, and the average time since treatment completion was approximately 3.85 (SD = 2.8) years. Participants most often reported unmet needs in the information and emotional health domains. They also described inadequate physical infrastructure (e.g., overcrowded clinic spaces) and minimal involvement of multidisciplinary providers. Despite these challenges, survivors expressed strong trust in physicians and a preference for physician-led care. Telehealth was viewed as a potential solution to reduce access barriers and improve continuity of care. **Conclusions:** Guided by the SUNS framework, this study identified significant unmet needs among colorectal cancer survivors in Thailand, particularly in health information, psychological support, and care system infrastructure. Strengthening health information delivery, establishing dedicated survivorship clinics, and exploring scalable care models supported by telehealth could help bridge these gaps and promote more equitable survivorship care in low-resource settings.

## 1. Introduction

Recent medical advancements in the early detection and treatment of colorectal cancer (CRC) have significantly improved overall cancer survival rates worldwide, particularly in high-income countries (HIC). In Western nations, the 5-year survival rates for CRC range from 62% to 71% [1]. In contrast, lower survival rates remain an issue in low- and middle-income countries (LMIC) like Thailand, where the rates have only modestly increased from 42% to 46% over the past two decades [2]. As survival rates rise, there is growing concern about the adequacy of post-treatment care for CRC survivors.

Globally, CRC survivors face long-term challenges after treatment completion, including physical, psychological, and socioeconomic concerns [3,4]. With inadequate support from healthcare systems, unmet needs—defined as areas where survivors do not receive sufficient support, services, or resources to manage post-treatment issues—remain a persistent challenge among CRC survivors [5]. Luo and colleagues [6] synthesized the qualitative, quantitative, and mixed-method literature and reported unmet needs for post-treatment CRC survivors. While CRC survivors in different post-treatment periods identified varying unmet needs [7], the most common included help with fears of cancer recurrence, information on managing illness and side effects at home, emotional or psychological support and reassurance, and assistance with sexuality concerns [6]. Higher rates of unmet needs were found to be associated with younger age, female gender, higher education level, unmarried status, as well as higher levels of distress, anxiety, depression, and worse quality of life [6].

In the Asia-Pacific region, a multi-country study found that cancer survivors in LMICs, including CRC survivors, reported significantly higher levels of unmet needs compared to those in HICs (all *p* < 0.001) [8]. Thai survivors, in particular, reported mean unmet needs scores of 38, more than double those of Australian survivors (mean = 17). The most pressing unmet needs among survivors in LMICs were in the areas of psychosocial care and access to comprehensive cancer care [8]. A study conducted in Thailand found that 80% of cancer survivors reported at least one unmet need within the past month. The most frequently reported concerns were fear of cancer recurrence, the need for high-quality medical care, and access to local healthcare services when needed [9].

Although cancer survivors in Thailand report substantial unmet needs, structured survivorship care has yet to be fully implemented [10]. Current national cancer policies prioritize cancer prevention, early detection, treatment, and palliative care [11]. CRC patients are typically treated in tertiary hospitals or cancer centers, where nurses provide discharge planning before patients return home [12]. During follow-up, survivors usually receive ongoing care from surgeons, oncologists, and nurses in outpatient departments [10]. However, it remains unclear whether this approach sufficiently addresses the complex and evolving needs of CRC survivors or improves their long-term outcomes.

Several qualitative studies have explored CRC survivors’ experiences, but most of those studies focused on the consequences of CRC and its treatment. However, only two studies conducted in HIC contexts have described CRC survivors’ experiences with healthcare services received [3,4]. These studies suggested that CRC survivors felt guilty about seeking help from healthcare providers (HCP) and experienced difficulties contacting them when necessary. Also, CRC survivors felt overwhelmed by the shift in care responsibilities from the oncologist to themselves, resulting in feelings of abandonment during survivorship care [4]. Given the lack of formal survivorship programs in Thailand, it is unclear how well existing follow-up care addresses survivors’ needs—a gap this study seeks to fill.

The Survivors Unmet Needs Survey (SUNS) was systematically developed to identify and categorize the unmet needs of cancer survivors following the completion of treatment [13]. It outlines five domains that commonly define the survivorship experience: information, emotional health, financial concerns, access and continuity of care, and relationships. The SUNS has been validated across diverse cancer populations and healthcare settings, making it broadly applicable to understanding survivorship across cultural and healthcare system contexts [14]. It has been used internationally, including in cross-cultural studies, highlighting its relevance in LMICs, where formal survivorship care is often limited [15,16]. Although the SUNS is a quantitative instrument, its domain structure offers an actionable framework for exploring unmet needs qualitatively. In this study, the SUNS was used to guide the analysis, allowing for the consideration of known contextual factors that shape unique survivorship experiences and contribute to a cohesive global discourse in survivorship care.

This study qualitatively explores the experiences of CRC survivors in Thailand through the five domains of the SUNS framework to understand survivors’ post-treatment needs and priorities. The study findings have important implications for identifying and addressing the unmet needs of CRC survivors in Thailand.

## 2. Materials and Methods

### 2.1. Study Design

This study used a qualitative descriptive design [17,18], appropriate for practice-oriented nursing and healthcare research [19,20]. This approach allowed for straightforward descriptions of CRC survivors’ experiences and unmet needs in the Thai context, supporting practical application in clinical interventions and service planning [19,20]. Reporting followed the 32-item Consolidated Criteria for Reporting Qualitative Research (COREQ) checklist [21].

### 2.2. Participants and Setting

The study participants were CRC survivors who attended follow-up appointments at a surgical outpatient department of a public tertiary university hospital in Bangkok, Thailand’s capital, between July and September 2022. This publicly funded university-affiliated hospital is one of Thailand’s leading academic medical centers. It provides specialized care, trains medical professionals, conducts research, and serves as a key referral hub for complex cases. With a longstanding reputation for excellence in cancer care, the hospital is recognized nationally for its advanced medical equipment, highly trained specialists, and central role in national cancer treatment and education.

The inclusion criteria were as follows: (1) aged 18 years or older; (2) had been diagnosed with CRC stage I, II, III, or IV; and (3) had completed curative treatment for CRC at least 1 year previously. We excluded individuals who had further treatment pending or were receiving palliative/end-of-life care.

Several methodological approaches have been employed to justify sample sizes in qualitative research, with several factors influencing these decisions [22]. Existing literature indicates sample sizes sufficient to reach saturation in interview-based studies typically range from 5 to 24 participants [23]. Accordingly, we recruited 24 participants, representing the upper bound of this range. This decision was made to maximize the likelihood of achieving both code saturation—the point at which no new themes emerge—and meaning saturation—when a comprehensive understanding of the themes is developed [24].

### 2.3. Recruitment

Using purposeful sampling, participants were chosen to achieve maximum variation in their main HCPs, sociodemographic characteristics, and clinical status. Potential participants were initially identified using patient follow-up appointment lists. Due to COVID-19 restrictions, potential participants were recruited via telephone within 7 days of completed follow-up visits. During the call, the first author, C.D., introduced the study, screened for eligibility, and requested permission to continue communication through the Line application. If permission was granted, participants were sent an electronic information sheet and consent form via Line for their review and records. Eligible individuals who agreed to participate in the study were scheduled for a subsequent online interview.

### 2.4. Data Collection

Data were collected using semi-structured individual interviews. The interview guide was developed based on prior related studies and input from the research team. It consisted of broad, open-ended questions accompanied by optional prompts used to facilitate deeper exploration of participants’ responses. The guide was pilot-tested and refined before data collection to ensure participant understanding of questions and to determine the appropriate sequence and approximate length of the interview. The final semi-structured interview questions are presented in Table 1.

The first author, C.D., conducted all interviews online via the Line application. C.D. is a Thai native who formerly worked as a registered nurse in the study setting and has clinical and research experience with the CRC population. C.D. audio-recorded the interviews, which lasted from 60 to 90 min, averaging 78 min. C.D. also took concurrent field notes. After each interview, the participants received 300 Thai Baht (approximately 8.50 USD) as compensation for their time.

Before the interviews, a brief telephone questionnaire was administered to collect participants’ sociodemographic and cancer-related characteristics, such as age, gender, education level, marital status, residence, cancer stage, treatment history, and time since treatment completion.

### 2.5. Data Analysis

The audio recordings of the interviews were transcribed verbatim in the Thai language. The transcripts were checked for accuracy, and any typographical errors were corrected. ATLAS.Ti Web version 23 was used for data management.

We employed a hybrid approach to qualitative content analysis, combining inductive coding with a deductive framework to guide interpretation. This approach follows the method proposed by Elo and Kyngäs [25], which involves data preparation, organization, and reporting. During the preparation phase, to obtain a holistic understanding, C.D. familiarized himself with the data by repeatedly listening to the interview recordings during the transcription process, studying all of the transcripts multiple times, and making notes of important points.

In the organization phase, C.D. coded transcripts line-by-line using an open coding approach to identify potential codes. These initial codes were then sorted into a structured coding list. Field notes were reviewed alongside the transcripts to help contextualize participants’ responses and enrich the interpretation of codes. The codebook was initially developed by C.D., then reviewed and finalized by all other researchers (S.A., R.J., G.G., C.I., A.M.).

The analysis was conducted by C.D., who re-examined the data to identify connections among codes and then deductively organized them into categories aligned with the SUNS domains. While coding was conducted inductively, the SUNS framework guided the final categorization, providing a structured lens for interpretation. Although we remained open to the emergence of new themes beyond the framework, all categories identified from the data were meaningfully organized within the five SUNS domains.

Multiple research team members were involved throughout the process. It was not feasible to assign independent coding to multiple researchers due to Thai language limitations among the team members. Instead, we adopted a collaborative approach involving regular peer debriefing and team discussions to enhance analytic rigor and reduce bias. The initial coding scheme and emerging categories were reviewed in these sessions. Based on team feedback, C.D. revised the coding, reassessed the data, and refined the categorization. This iterative process continued until all of the researchers (C.D., S.A., R.J., G.G., C.I., A.M.) reached consensus on the final coding framework and interpretation of findings. This analytic strategy was appropriate and aligns with established qualitative research practices that support sole coding when the researcher demonstrates prolonged engagement with the data, contextual understanding, and employs multiple strategies to ensure rigor [26,27]. A preliminary description of the results was then developed, with each category supported by illustrative quotations from the interviews.

All interviews were conducted, audio-recorded, transcribed, and analyzed in Thai. Quotations were later translated into English for reporting, following a rigorous translation verification process. C.D. translated quotations from Thai into English. Assistance in the translation process was obtained from an English-language editor with experience in scientific writing in nursing science. Subsequently, two bilingual doctoral students in nursing science who were not members of the research team reviewed the translated quotations for accuracy. Any discrepancies identified were addressed by C.D., who made minor revisions to the translation to achieve the participants’ intended meaning. All of the researchers (C.D., S.A., R.J., G.G., C.I., A.M.) reviewed the English language quotations to further confirm alignment with categories. The final analysis presented in this paper is accompanied by the most representative quotations to illustrate each category. Finally, SPSS version 26 (SPSS Inc., Chicago, IL, USA) was used to perform a descriptive analysis of the data on participant characteristics collected using the brief questionnaire.

### 2.6. Rigor

To ensure the trustworthiness and rigor of the study findings, several established qualitative techniques were employed to enhance credibility, dependability, confirmability, and transferability [19,28]. Credibility was achieved using prolonged engagement with the interview data, specifically the verbatim transcription of the interview recordings, multiple re-readings of the interview transcripts, and thorough translation of participant quotations into English. Moreover, rapport was developed with the participant before each interview to establish trust. The research team was selected to include various health disciplines and areas of expertise. Consequently, the team triangulated multiple viewpoints that subsequently informed the interpretation of results. Also, C.D. held regular peer debriefings with doctoral students in other health disciplines to obtain additional feedback on the interpretation of the data.

To enhance dependability and confirmability, C.D. engaged in reflexivity, composed memos, and created an audit trail. The research team members reviewed, audited, and confirmed the coding process and results in regular meetings. Furthermore, direct quotations were extracted from the interview transcripts to illustrate and support the results.

Finally, strategies employed to improve the transferability of the study results included applying a purposeful sampling approach and preparing thick descriptions of the study context, procedures, and findings.

### 2.7. Ethical Considerations

The Institutional Review Boards of the University of Illinois Chicago (Protocol #2022-0157) and the regional hospital in Thailand (Protocol #130/2565) approved the study. All participants were informed about the study’s aims, procedures, and their rights, including the voluntary nature of participation and the assurance that declining would not affect their clinical care. A written information sheet was shared via Line prior to the interview. Because data collection occurred remotely using Line, written consent was not feasible. Instead, C.D. reviewed the consent form with each participant verbally before the interview and documented oral consent. This approach is consistent with standard ethical guidelines for remote research and was approved by both IRBs. Participants also provided oral permission for audio recording.

The confidentiality and anonymity of all participants were secured throughout the study. Each participant was assigned a unique identification number, and all direct identifiers were removed from transcripts and study materials. Electronic data were stored on password-protected, encrypted drives accessible only to essential research personnel. Identifiers were destroyed at the earliest possible time, per the IRB-approved protocol. Findings were reported in aggregate to prevent the identification of individual participants.

## 3. Results

### 3.1. Participant Characteristics

Table 2 presents the participants’ sociodemographic and cancer-related characteristics. Among the 24 participants, the average age was 57.2 years. At the time of the interviews, the average time elapsed since the end of primary treatment was 3.9 years. Most participants were male (57.2%), reported being married (66.7%), and lived in the central part of Thailand (58.3%). Also, most had at least a college degree (41.7%), and were currently employed (41.7%). More than 60% of participants were diagnosed at a later stage, with 45.8% at stage III and 16.7% at stage IV. All participants had undergone surgery, and 70.8% had received combination therapy (i.e., surgery and chemotherapy). Additionally, 45.8% reported having a colostomy.

### 3.2. Qualitative Findings

Table 3 outlines categories that emerged from interviews with CRC survivors. Based on the five domains of the SUNS: (1) information, (2) emotional health, (3) financial concerns, (4) access and continuity of care, and (5) relationships, a total of 15 categories were identified. These categories reflect the complex and interrelated challenges experienced by post-treatment Thai CRC survivors and capture the specific needs within each domain.

#### 3.2.1. Information

##### Inadequate and Inconsistent Survivorship Information

Participants reported receiving insufficient or conflicting information about survivorship, particularly regarding diet and self-care practices. Survivors often turned to peers, the internet, or other informal sources due to the lack of consistent guidance from HCPs, which led to confusion and uncertainty. Participants also noted that available educational materials were too generic and did not address their nuanced or individualized needs during survivorship.

##### Communication Barriers with Healthcare Providers

Participants expected their HCPs to proactively initiate discussions and provide essential information covering all aspects of survivorship care. However, they often experienced brief consultations focused primarily on test results and physical health concerns. Several participants reported feeling rushed, anxious, or hesitant during follow-up visits, which hindered their ability to process information or engage in meaningful dialogue with their HCPs. They also expressed uncertainty about what concerns they should express, what types of questions they could ask, or how to ask them.

##### Medical Jargon

Obtaining and understanding information specific to one’s diagnosis and treatment has been shown to play a significant role in patient outcomes, including treatment adherence and satisfaction with care. In Thailand, medical information is typically provided directly to patients through medical records. However, participants in this study reported considerable difficulty understanding their medical records, which were often written in English and contained complex medical terminology. They expressed a strong need for simpler, clearer, and more specific verbal explanations from their healthcare providers regarding test results and their survivorship status.

The lack of clear, consistent information and communication barriers with healthcare providers not only left participants confused about their care but also contributed to heightened anxiety and emotional distress. Survivors’ struggles with medical jargon and limited guidance on survivorship needs were closely tied to feelings of uncertainty and vulnerability. These informational gaps created emotional challenges.

#### 3.2.2. Emotional Health

##### Psychological Distress

Participants reported experiencing ongoing psychological distress. A primary psychological concern was fear of cancer recurrence. Many participants reported persistent anxiety that their CRC might return or that cancer could develop in other parts of their bodies. This fear was often elevated by hearing about others’ experiences with recurrence, especially while awaiting their own surveillance test results. Despite a provider’s inability to accurately predict an individual’s risk of recurrence, the absence of clear and direct communication from HCPs regarding their recurrence risk contributed to uncertainty and emotional distress.

##### Lack of Psychological Support

Despite experiencing emotional challenges, participants reported receiving limited psychological support and few opportunities to express their concerns during follow-up care. Their emotional needs were often unmet, as the focus of care remained centered exclusively on the biomedical issues rather than psychosocial well-being.

In summary, participants experienced significant emotional distress—particularly related to fear of recurrence—but reported limited psychological support, with provider interactions primarily focused on physical symptoms rather than emotional well-being.

#### 3.2.3. Financial Concerns

##### Mixed Financial Challenges

Participants reported experiencing a range of financial challenges during the survivorship phase. While national health insurance was recognized as a key facilitator that alleviated many direct treatment-related expenses, out-of-pocket costs for certain medications and medical supplies necessary for ongoing survivorship care remained a significant concern. Participants were unsure about the extent of their insurance coverage. Indirect costs, such as transportation and accommodation expenses associated with follow-up visits, were particularly burdensome for those residing in rural areas and contributed to sustained financial strain.

##### Employment-Related Challenges

Bowel-related complications created significant barriers to employment. Participants reported difficulty returning to work, needing to reduce responsibilities, role changes, or leaving their jobs entirely due to defecation issues and feared potential stigma or discomfort in the workplace. These challenges affected their financial stability. Participants also noted a lack of support or guidance from HCPs regarding employment and return-to-work planning.

In summary, participants faced persistent financial concerns related to both employment disruptions and ongoing out-of-pocket expenses for survivorship care, compounded by limited guidance from providers and inconsistent insurance coverage.

#### 3.2.4. Access and Continuity of Care

##### Cumbersome and Time-Consuming

Participants described accessing survivorship care as burdensome and time-consuming. Routine follow-up visits often required them to arrive at the treatment center early to complete multiple administrative procedures. These included navigating several processes and performing tasks at various stations and buildings. Long waiting times were frequently reported. Participants also described having only brief consultations with physicians. Additionally, participants residing in rural areas faced further challenges, including long-distance travel, often beginning the night before, and the need for overnight accommodation near the hospital to ensure timely attendance.

##### Inadequate Infrastructure

Participants identified limitations in hospital infrastructure as an additional barrier to receiving follow-up care. Several aspects of the physical treatment environment were described as contributing to patient frustration and discomfort, and in some cases undermining their sense of privacy. Commonly reported concerns included overcrowded clinic spaces, insufficient seating, inadequate restroom facilities, low-volume announcements, and limited parking availability. These environmental challenges were perceived to hinder the care experience and add to the overall burden of survivorship care.

##### Lack of Multidisciplinary Support

Participants identified the absence of a multidisciplinary care team as a barrier to survivorship care. They reported that their survivorship care was primarily physician-led, with minimal involvement from nurses and no engagement with other healthcare professionals. This limited approach left gaps in addressing broader survivorship needs, such as health education, psychosocial support, and lifestyle guidance. Participants expressed a desire for greater nursing involvement, particularly in areas related to health promotion and patient education.

##### Effective Scheduling of Appointments

Participants described the hospital’s scheduling system as a key facilitator of survivorship care. Follow-up appointments were typically arranged during prior visits, with scheduling processes reported as flexible and patient-centered. Participants appreciated the clarity of written appointment documentation and valued the convenience of reminders via mobile applications or telephone calls. These systems supported timely and consistent engagement in follow-up care.

##### Commitment to Continued Care

Despite experiencing challenges, participants demonstrated a strong commitment to attending follow-up appointments. They expressed trust in their physicians and viewed surveillance visits and testing as critical to monitoring their health and detecting recurrence. This intrinsic motivation served as a facilitator of continued engagement in survivorship care.

##### Telehealth as a Potential Solution

Participants identified multiple barriers to survivorship care, including travel burdens, long waiting times, and brief consultations. In light of these challenges, they were asked to reflect on whether telehealth could help address some of these issues. Although participants had no experience with telehealth, many expressed openness to its integration into survivorship care, particularly as a strategy to improve access and reduce the time and financial burden associated with hospital visits. Telehealth was viewed as appropriate for communicating surveillance test results, addressing minor concerns, and serving patients who are comfortable with technology. However, participants emphasized the limitations of virtual consultations in providing comprehensive physical assessments.

In summary, logistical burdens, limited infrastructure, and gaps in multidisciplinary support shaped access to and continuity of care, yet participants remained highly motivated to attend follow-ups and welcomed telehealth as a potential means to ease access barriers.

#### 3.2.5. Relationships

##### Relational Trust in Cancer Care

Participants expressed a strong sense of trust in the hospital and HCPs. This trust was rooted in the hospital’s longstanding reputation as a leading academic medical center, its specialization in cancer care, and access to advanced medical equipment. Their confidence was further reinforced by positive firsthand experiences during treatment and successful outcomes. Participants also highlighted the dedication and compassion of HCPs as central to their trust in care. They described providers as caring, considerate, and committed to patient well-being.

##### Preference for Physician-Led Survivorship Care

Stemming from the trust outlined above, participants consistently expressed a strong preference for receiving survivorship care from physicians, particularly those involved in their cancer care since diagnosis at the same treatment center. They perceived physicians as the most qualified to interpret test results, monitor for recurrence, and provide reliable health promotion advice. Continuity with the same physician was considered essential, as participants felt that their ongoing care benefited from the physician’s familiarity with their medical history and cancer journey.

Participants expressed concerns about transitioning to other healthcare settings, fearing they would have to restart the care process, reestablish trust, and potentially become lost in the care transition. Participants were reluctant to receive follow-up care from nurses. Nurses were perceived as having limited roles, experience, and expertise in providing comprehensive survivorship care, contributing to a general lack of trust in nurse-led survivorship care.

In summary, participants’ relationships with HCPs were characterized by deep trust in the cancer care team and a strong preference for physician-led follow-up, reflecting the value placed on provider expertise, continuity of care, and institutional reputation.

## 4. Discussion

This qualitative study is among the first in Thailand to examine CRC survivors’ post-treatment experiences and needs. Participants reflected positively on the care they received during active treatment, often expressing deep gratitude for having survived cancer. This sense of gratitude contributed to a strong, enduring trust in their HCPs, which shaped their ongoing engagement with the healthcare system during survivorship.

Despite these areas of satisfaction regarding their acute care, participants reported various ongoing post-treatment challenges and unmet needs that align closely with the five domains of the SUNS framework: information, emotional health, financial concerns, access and continuity of care, and relationships. These concerns indicate a gap between survivors’ long-term needs and the scope of services typically provided during routine follow-up visits. Consistent with prior global studies [4,6], participants reported insufficient survivorship-specific information, particularly around diet and self-care practices. Emotional distress was also prevalent, yet psychosocial support was rarely offered as part of routine follow-up. Financial toxicity emerged as a significant concern, even within a publicly funded healthcare system, mirroring global evidence that direct and indirect costs continue to burden survivors [29,30]. Challenges in care access—including long travel distances, lengthy wait times, and brief consultations—echo issues identified in other LMICs [31].

An important observation was the communication barriers between CRC survivors and their HCPs. This finding likely reflects cultural values and healthcare system dynamics unique to the Thai context. The concept of *Kreng-jai*—a deep-seated reluctance to inconvenience or challenge authority [32]—may discourage survivors from posing questions or seeking additional support, particularly during follow-up visits. In highly hierarchical clinical encounters, deference to physicians may further discourage active help-seeking, especially for psychosocial or informational concerns [33]. From a behavioral perspective, the Health Belief Model frames these sociocultural expectations as perceived barriers that lower the likelihood of acting on unmet needs unless clinicians provide explicit cues and permission to speak up [34]. While such dynamics are not exclusive to Thailand, recognizing these cultural patterns is essential for developing culturally responsive survivorship care models that both invite patient questions and normalize shared decision-making, thereby empowering survivors in hierarchical, LMIC settings such as Thailand. These approaches have shown promise in other contexts [35] and could be adapted and tested for feasibility and acceptability in Thai survivorship care.

The male-majority sample reflects national trends in CRC incidence, which are higher among males in Thailand [36]. This male predominance may have disproportionately shaped the findings, as men are generally less likely than women to report psychosocial distress or seek informational support during cancer survivorship [37]. Cultural expectations of masculine stoicism may further temper disclosure of unmet needs [38]. Consequently, concerns more commonly voiced by women—such as body-image issues or caregiving burden—may be under-represented [39]. Because survivors’ unmet needs appear to vary with intersecting factors such as gender, geographic location, and socioeconomic status [40], future research should adopt an explicit intersectional lens to clarify how these overlapping identities shape survivorship experiences.

Globally, efforts have been made to improve the quality of survivorship care through structured models and policy initiatives. In 2005, the IOM defined cancer survivorship as a distinct phase of the cancer care continuum and emphasized the need for comprehensive post-treatment care, including surveillance for recurrence and new cancers, management of late effects, psychosocial support, and health promotion. Delivering this level of care requires coordination among multidisciplinary HCPs [41].

Although countries show significant variations in their healthcare capacity and cancer control planning, implementation of quality survivorship care is deficient worldwide [42,43,44,45]. A substantial reason for this gap is that survivorship care is often delivered within traditional follow-up frameworks, prioritizing medical surveillance over comprehensive support. As a result, these services may fail to address survivors’ full array of needs. This also appears to be the case in Thailand, where study participants reported that post-treatment care was primarily limited to periodic physician visits, with few additional support services available. These findings are consistent with global literature describing survivorship care as fragmented and insufficiently person-centered [42,43,44,45].

While survivorship care models have become more prevalent in high-income countries, Thailand is still in the early stages of establishing formal programs. Thailand’s cancer control efforts have primarily focused on prevention, early detection, treatment, and palliative care, with limited policy attention directed toward survivorship [11]. Although a range of cancer care services are available at tertiary hospitals and cancer centers [10,12,46], the study suggests that the current follow-up care may not fully incorporate key components of high-quality survivorship care outlined by the IOM.

### 4.1. Implications

This study offers important insights for strengthening survivorship care in Thailand and other LMICs. The findings underscore the urgent need to develop affordable, context-sensitive survivorship care infrastructure in health systems traditionally focused on prevention and acute treatment. The unmet needs identified across the SUNS domains—including inadequate information, limited emotional support, and barriers to access and continuity of care—directly inform the recommendations for improving survivorship services. Drawing on these findings, we propose a policy roadmap tailored to LMIC contexts to guide the development and implementation of comprehensive survivorship care (Table 4).

#### 4.1.1. Institutionalize Survivorship Care as a Distinct Phase of Cancer Care

Our findings of fragmented follow-up care underscore the need, also emphasized in the literature, for policy-level recognition of survivorship as a distinct phase of care. However, this phase remains underdeveloped in national cancer strategies. As part of institutionalizing survivorship care, healthcare systems should routinely assess survivors’ holistic needs and ensure access to appropriate support services. Given their longitudinal role in care delivery, nurses are well-positioned to lead these assessments. Several validated instruments can facilitate this process, including the Cancer Survivors Unmet Needs, which has been translated and validated in Thai, showing strong reliability and content validity [47]. Incorporating such tools into routine care pathways offers a feasible and cost-effective strategy for identifying and addressing the diverse needs of CRC survivors in Thailand.

#### 4.1.2. Develop Nurse-Led Survivorship Care Models

While CRC survivors in this study preferred physician-led follow-up due to trust and continuity, expanding the role of nurses in survivorship care is necessary and feasible. In Thailand, nurses already play a central role in patient education, psychosocial support, and care coordination. Leveraging this existing scope of practice aligns with their professional training and responsibilities, particularly within oncology settings. More formally integrating nurses into survivorship care—through structured education, policy support, and interdisciplinary collaboration—could help build survivor confidence in nurse-led models and enhance acceptance over time.

Global evidence supports the effectiveness of nurse-led survivorship programs in delivering follow-up care, managing symptoms, and enhancing patient satisfaction [48], especially when nurses are introduced as trusted multidisciplinary team members. In Thailand, oncology nurses are ready to expand their roles, particularly in implementing survivorship care plans (SCP) [49]. While advanced practice providers, such as nurse practitioners, are central to survivorship care in HICs like the United States [50], similar functions can be achieved in LMICs through task-sharing models. Empowering Thai oncology nurses to assume leadership in survivorship care presents a scalable strategy to improve access, continuity, and quality of care in resource-constrained settings.

#### 4.1.3. Standardize and Implement Survivorship Care Plans

Participants’ confusion and lack of guidance highlight the need for formal survivorship care planning as recommended by existing literature [41]. Participants did not receive such plans, likely contributing to their informational gaps and uncertainty. SCPs summarize essential information, including diagnosis, treatments, follow-up schedules, and supportive care resources. They serve as a bridge between hospital-based treatment and long-term survivorship. Although evidence regarding the clinical outcomes of SCPs is mixed, research consistently shows that SCPs improve care coordination, clarify provider roles, enhance survivor satisfaction, and aid in information retention [51,52,53].

A recent study found strong support for SCP adoption among Thai oncology nurses, who recognized the value of each component and expressed their willingness to lead SCP preparation and delivery [49]. Using plain, lay-friendly language in SCPs can improve survivor–provider communication, particularly when addressing complex follow-up instructions [52,54]. While some HICs have revised their requirements, international organizations continue to endorse SCPs as a cornerstone of survivorship care [41,55]. In LMICs, simplified SCP templates translated into local languages and delivered electronically or during treatment discharge could enhance accessibility, cost-efficiency, and cultural appropriateness.

#### 4.1.4. Enhance Health Information Delivery

Because participants reported a significant lack of information and numerous communication barriers, which hindered their ability to manage their health and daily life, we recommend enhancing health education efforts to better support survivors. Creating culturally relevant and literacy-sensitive survivorship resources is essential to empowering cancer survivors in LMICs. Improving health literacy has been linked to better outcomes, including decision-making, care navigation, medication adherence, and quality of life [56]. If implemented effectively, SCPs can function as an educational and communication tool. Prior research has shown that cancer survivors value SCPs written in plain language, as they support communication with healthcare providers and promote self-management [52]. To increase accessibility, mobile health platforms can disseminate survivorship content, deliver reminders, and summarize test results. These platforms offer a scalable, low-cost strategy for improving health literacy and empowering survivors with actionable guidance.

#### 4.1.5. Establish Survivorship Clinics and Decentralized Services

This study identified infrastructure limitations and centralized care processes as barriers to survivorship care. To address these challenges, we propose establishing dedicated CRC survivorship clinics at tertiary treatment centers as a critical step toward delivering comprehensive, multidisciplinary post-treatment care and strengthening survivorship programs. International models, such as the survivorship clinic at William Osler Health System in Canada, have successfully supported care transitions, managed symptoms, and reduced patient distress through structured survivorship clinics [57].

However, exclusive reliance on centralized care can create geographic and financial barriers, particularly for survivors in rural or underserved areas. A shared care model—where oncology specialists and primary care providers collaboratively manage survivorship care—offers a promising solution to address these challenges. Studies have shown that shared care models can improve patient satisfaction and ensure comprehensive management of cancer-related and general health needs [58]. Additionally, integrating telehealth services can support shared care by enabling virtual consultations and case reviews between specialists and local providers. Teleconsultations for stable surveillance can further enhance accessibility and continuity of care for survivors, regardless of location.

#### 4.1.6. Invest in Training and Capacity Building

In this study, participants reported minimal involvement of nurses in survivorship care, which may reflect gaps in knowledge and skills related to post-treatment needs. To address this, we recommend incorporating survivorship-focused content into nursing education and continuing professional development programs. Cost-effective training formats—such as online modules, short-course certifications, and on-the-job training—could expand access and equip healthcare providers with the competencies to deliver quality survivorship care. Investment is also needed in faculty development and academic infrastructure, including preparing educators, supporting oncology nursing program accreditation, and establishing mentorship systems [59,60]. Strengthening the oncology nursing workforce and expanding nurses’ roles in LMICs is essential. Capacity building efforts aligned with national workforce strategies could elevate nurses’ leadership in survivorship care.

### 4.2. Strengths and Limitations

This study addresses a critical gap in research and brings attention to an unexplored area of survivorship care. The study’s strengths include including participants with a wide range of sociodemographic characteristics (e.g., broad age range), clinical statuses, and variations in HCPs, which adds to the breadth of the survivors’ experiences and perceptions. However, this study involved only gender-binary individuals, with most identifying as male. Although qualitative research does not typically aim for generalizability, including non-binary or gender-nonconforming individuals could have enhanced the study’s comprehensiveness in reflecting diverse perspectives on survivorship care.

As one study limitation, because all interviews were conducted via Line audio calls instead of in person, as was intended, there was no way to observe participants’ non-verbal responses or to verify documents they referred to. Also, as participation in this study was voluntary, self-selection bias may have been present. Only one researcher performed the initial coding, which could introduce bias; however, team-based reviews and discussions of codes and themes helped mitigate this risk. Participants also did not review the study findings due to logistical constraints, which limited opportunities for member checking to validate interpretations. While many approaches were used to maximize the accuracy of the English translation of participant statements, the accuracy of translated transcript quotations may have been influenced by culture-related linguistic differences.

Overall, this study was limited to the experiences and perceptions of CRC survivors, so future studies are needed to explore the experiences and perceptions of family members and caregivers in navigating care for the survivors. Additional research should also be conducted among other types of cancer survivors. Furthermore, this study did not seek to differentiate between the experiences of CRC survivors according to their sociodemographic or clinical characteristics, which could have impacted participants’ experiences of survivorship care. Moreover, some categories may have been shaped primarily by insights from a small number of participants, raising the possibility that individual perspectives were overemphasized within certain themes. As a result, the ability to distinguish broadly shared experiences from more unique or context-specific views may be limited. While all of the categories that emerged from the data could be meaningfully organized within the SUNS framework, the use of a predefined structure may have constrained the identification of novel or culturally specific themes beyond the framework.

Finally, participants were enrolled in a single tertiary university hospital in the capital city of Thailand. This single-center design constrains the transferability of findings, as it limits the ability to capture variations in survivorship care across primary and secondary care levels or in other geographic regions of the country. Although this study was framed within the context of the Thai healthcare system, most of the findings and related implications may be transferable to other Asian and LMICs, especially those where CRC care is provided within similar healthcare systems. Nonetheless, the results should be interpreted cautiously before applying them to countries with substantially different hospital capabilities, healthcare systems, and cultures.

## 5. Conclusions

In conclusion, CRC survivors in Thailand face diverse unmet needs beyond clinical care, highlighting limitations of existing services. While based on a single-center study, these findings point to several priority actions to strengthen survivorship care in LMICs. Key actions include implementing systematic needs assessments and expanding survivorship services through dedicated clinics and greater oncology nurse involvement. These strategies, especially those that are telehealth-supported, could significantly improve access and equity in survivorship care in Thailand and similar settings. Future research should explore the implementation of these strategies and evaluate outcomes.

## Figures and Tables

**Table 1:** Semi-structured interview questions.

1.	What have your experiences been receiving follow-up care from healthcare providers since treatment completion? *Prompts:* - What types of services have you received from your healthcare providers? - Are you satisfied with the services you have received? Why/Why not?
2.	What has been helpful for you in receiving follow-up care from healthcare providers? *Prompts:* - What has made it easier for you to get the services you need? Please give an example of how this has made it easier. - Who has made it easier for you to get the services you need? Please give an example of how they have made it easier. - How has the hospital made getting the services you need easier? Please give an example of how this has made it easier.
3.	What obstacles have you encountered in obtaining follow-up care from healthcare providers? *Prompts:* - What has made it harder for you to get the services you need? Please give an example of how this has made it more complicated. - Who has made it harder for you to get the services you need? Please give an example of how they have made it harder. - How has the hospital made it harder for you to get the services you need? Please give an example of how this has made it harder.
4.	What recommendations do you have for healthcare providers and the hospital to help them provide follow-up care? *Prompts:* - What does the hospital need to improve to provide better follow-up care services that you need? - What changes would improve the overall follow-up care services you need?
5.	Can you tell me about your preferences for follow-up care? *Prompts:* - By whom, where, and how should follow-up care be provided?

**Table 2:** Participant characteristics (n = 24).

Characteristic		N (%)	Mean (SD; Range)
Age (years)			57.2 (10.90; 20–78)
Years since treatment end (years)			3.9 (2.84; 1–11)
Gender			
	Male	14 (58.3)	
	Female	10 (41.7)	
Marital status			
	Married	16 (66.7)	
	Single	4 (16.7)	
	Separated/divorced	2 (8.3)	
	Widowed	2 (8.3)	
The Thailand region of residence			
	Central	14 (58.3)	
	Eastern	5 (20.8)	
	Western	3 (12.5)	
	Northeastern	1 (4.2)	
	Southern	1 (4.2)	
Education level			
	Elementary school	6 (25.0)	
	High school	8 (33.3)	
	College/university	10 (41.7)	
Employment status			
	Employed	10 (41.7)	
	Unemployed	6 (25.0)	
	Retired	8 (33.3)	
Stage of cancer at diagnosis			
	1	5 (20.8)	
	2	4 (16.7)	
	3	11 (45.8)	
	4	4 (16.7)	
Cancer treatment			
	Surgery only	7 (29.2)	
	Surgery + chemotherapy	10 (41.6)	
	Surgery + radiation therapy	1 (4.2)	
	Surgery + chemotherapy + radiation therapy	6 (25.0)	
Colostomy			
	No	13 (54.2)	
	Yes	5 (20.8)	
	Reversed	6 (25.0)	

Abbreviations: N, numbers; SD, standard deviation.

**Table 3:** Domains of the Survivors Unmet Needs Survey, categories derived from the interviews, and participant quotations.

Survivors Unmet Needs Survey	Categories	Participant Quotations
Information	Inadequate and inconsistent survivorship information	“I talk to friends who have the same disease about which foods can or cannot be eaten. Red meats, such as pork and beef—they say that these kinds of food should be avoided. But the doctor says that I can eat anything. … I want to know what I can really eat. I also search for information on the internet. There are several opinions about foods; I don’t know which one I should follow. … There is no information from the hospital about what to eat, like no information on paper. … I am so confused.” (CRC-16) “There are materials about how to perform self-care…but it is like general knowledge. Some information is related to me, but some is not.” (CRC-04)
	Communication barriers with healthcare providers	“The doctor will ask whether I have any abnormalities. … I don’t know what to ask. … I can’t think off the top of my head. … I don’t know what I should ask the doctor, and I don’t know how to ask.” (CRC-12) “After I go inside [the examination room] and the doctor tells me about my test results, I forget about the questions that I prepared. … I don’t know what to ask. The doctor decides what to tell me. … Also, I am hesitant to ask questions because too many patients are in line after me.” (CRC-22)
	Medical jargon	“It is sometimes written in English, which I can’t understand. It also uses medical terms that I don’t understand. Better not look at it; letting the doctor explain it to me is better.” (CRC-05)
Emotional health	Psychological distress	“I have seen my neighbors have cancers and complete treatment, but the cancers recur at other sites. Consequently, I am worried that I will have cancer again. … Every time I go for follow-up, while I am waiting to see my doctor, I think about how my test results will turn out. When I hear from my doctor that everything is good, I feel relieved. But when I get home, I again think about whether cancer will come back. … I want my doctor to frankly tell me whether cancer will come back or not. I can’t get over it because my doctor hasn’t said [this].” (CRC-18)
	Lack of psychological support	“The doctor reviews the x-ray computer results and asks whether I have abdominal pain or mucus in my stools. If I don’t have any symptoms, we will see each other again next year. … My doctor rarely spends time discussing psychological concerns. … Mostly, he discusses symptoms.” (CRC-15)
Financial concerns	Mixed financial challenges	“The expenses are high, like travel costs. Each visit costs over 1000 Baht. … I used to buy the good-quality ones, but now I have to buy the ones that cost 50 Baht a box because I just can’t afford the better ones. I wanted to know why somethings under the civil servant medical benefit scheme can’t be reimbursed. … I’ve brought it up a few times, but maybe the nurses think it as a personal issue and just kind of listen. … I ask the nurse why I still have to pay out-of-pocket. … But I’ve never said straight out that I can’t afford it.” (CRC-09) “If I buy these kinds of supplies, I can’t get reimbursed. They are not covered, I have to pay for them myself. … If I take my own car, each trip costs around 5000–6000 Baht. And now, with gas prices being so high, I try to save, sometimes I take a bus instead. If I have to stay overnight, it ends up costing more than 7000 baht.” (CRC-21)
	Employment-related challenges	“I was worried about whether I could return to work. But when my sick leave was over, I had to go to work anyway, but I am still worried…because I have a colostomy. … They [HCPs] don’t ask whether I am okay or have difficulties with my daily life and work.” (CRC-15) “I couldn’t go to work. I had to stay home and use my savings. If I go to work, which involves shipping, I have to serve people at the front counter. When I need to poo, I have no chance of making it to the bathroom on time. That’s why I quit my job and stay home to take care of myself.” (CRC-23)
Access and continuity of care	Cumbersome and time-consuming	“I have to leave early, about 5 am. … When I arrive at the hospital, I need to register for my national health insurance. Then, I have to register and get in the queue for the blood tests and then go to another building, 3rd floor. … After that, I need to register for my visit; then my queue number will show up. … I get my blood tests done around 7 am, and I need to wait until 11 am to see my doctor…for approximately 10 min.” (CRC-01) “On the day of travel, I have to leave my home at 8 pm to wait for the bus because I don’t live in the city. Sometimes, I take a car…my son or husband will drive, and we will rent a room in the [hospital name] area. … I always get there early. … I am afraid that if I am late, I will lose my appointment or have to wait much longer.” (CRC-21)
	Inadequate infrastructure	“The clinic space is small, but there are so many patients. The announcements’ volume is quite low, sometimes I can’t hear. … It is burdensome when going to the bathroom…it is messy.” (CRC-04) “It is difficult to find parking. Sometimes I need to drive around for half an hour to find a parking space. Examination areas are quite crowded, especially in the morning when there are numerous patients visiting the hospital.” (CRC-02)
	Lack of multidisciplinary support	“Enterostomal therapy nurses who provide care and advice about colostomy care are good. … But I rarely interact with other nurses because they have nothing to do with my follow-up. … I think there should be [patient] education sessions, like having patients go into a room, and nurses teach them about how to monitor for abnormal signs, what to eat, and how to make notes to show to the doctors when having follow-up.” (CRC-23)
	Effective scheduling of appointments	“My doctor tells me to see him again in 3 months. Then the appointment will be scheduled at the appointment counter. … Last month, I had COVID, so I had to reschedule…which was not complicated. When I told them, they just gave me a new appointment. They asked which date worked best for me that matched my doctor’s schedule.” (CRC-01) “On the appointment paper, it is clearly indicated when the appointment date is, and which tests need to be done. … I use the [hospital name’s] mobile application, and the appointment notifications pop up.” (CRC-23)
	Commitment to continued care	“I come every time, never miss my appointments. … Whatever the doctor says, I will follow him. I obey the doctor. … I want to continue [the follow-ups] because I want to know whether there is any abnormality.” (CRC-03) “I always come for appointments. It is good to see my doctor and get my blood tested…. The doctor says that I have to be examined to see whether the cancer returns or spreads to other body parts. … I want to do that because I want to know whether I have a chance of having something else or anything is abnormal.” (CRC-08)
	Telehealth as a potential solution	“I have never used telehealth, but I think it would be good if it is available. … If they don’t have symptoms, it is okay [to use telehealth], especially for patients who live far away in other provinces and have difficulty travelling to the hospital. … Now that patients are better at using technology and smartphones, they would have reasonable access to telehealth.” (CRC-04) “I think it is okay to use video calls to notify patients of test results, and it would decrease the number of patients in the hospital. For example, after I get my blood test done, I could go back home immediately. I don’t need to wait for the test results. Then the doctors will tell me about the results via a video call. … But if I need to get a CT scan or [physical] examination, I think it is necessary [to go to the hospital].” (CRC-20)
Relationships	Relational trust in cancer care	“I want to continue my follow-up here because I trust this hospital. … This is like a doctor market. I notice that they always rush to patients to provide care. … Doctors put their hearts into treating patients…and are caring, including nurses. I really adore them. Also, there is sufficient medical equipment. As it is one of the medical schools, I am confident in my doctors. I am confident because of the reputation of the hospital, which has been established for more than 100 years. Everyone who comes to the [hospital name] would say this hospital is excellent.” (CRC-04) “They are the best. I think if I had gone to [another hospital name], I would have died already. … I am confident and trust them 100%…because they treat me well, cured [my cancer], and helped me survive until now. I am so proud and happy that they continue to take care of me.” (CRC-18)
	Preference for physician-led survivorship care	“I am not going [to follow up at different hospitals] because I might need to start over with registering my medical history. … It is better to follow up with the doctors who have treated me because they have known me from the beginning. … I want to follow up with doctors rather than nurses…because if there are any problems, the doctors could better explain my test results and better advise me about treatment.” (CRC-02) “I want to follow up with my regular doctors because they have treated me from the beginning and knows my history. … Nurses might be able to tell me if they read the information, but I trust the doctors more…because the doctors have more education and experience than nurses…and they have different expertise. … The doctors know more, study more, and can better explain and advise me.” (CRC-22)

**Table 4:** Policy implications for strengthening colorectal cancer survivorship care in low- and middle-income countries.

Policy Action	Recommended Strategies
Institutionalize survivorship care as a distinct phase	- Integrate survivorship into national cancer control plans - Routinely assess holistic needs using validated tools - Ensure access to multidisciplinary support services - Engage nurses to lead survivorship needs assessments
Develop nurse-led survivorship care models	- Expand nurses’ roles in follow-up care, education, and care coordination - Support nurses in leading SCP implementation - Implement task-sharing models to address workforce shortages
Standardize and implement survivorship care plans	- Develop simplified SCP templates in local languages - Use plain, accessible language for SCP content - Deliver SCPs at discharge or electronically
Enhance health information delivery and health literacy	- Create culturally appropriate, literacy-sensitive educational resources - Use SCPs as tools for patient education and communication - Leverage mobile health platforms to disseminate survivorship content and test results
Establish survivorship clinics and decentralize services	- Establish CRC survivorship clinics at tertiary care centers - Implement shared care models between specialists and primary care providers - Use telehealth and teleconsultation to extend access and reduce access barriers
Invest in training and capacity building	- Incorporate survivorship care into nursing curricula - Offer accessible training formats (e.g., online modules, short courses) - Develop faculty capacity and support academic infrastructure

Abbreviations: CRC, colorectal cancer; SCP, survivorship care plans.

## Data Availability

The dataset generated during and/or analyzed during the current study is available from the corresponding author upon reasonable request.

## Abbreviations

The following abbreviations are used in this manuscript:

CRC	Colorectal Cancer
HCP	Healthcare Providers
HIC	High-Income Counties
IOM	Institute of Medicine
LMIC	Low- and Middle-Income Countries
SCP	Survivorship Care Plans
SUNS	Survivors Unmet Needs Survey
